# Risk factors for pulmonary complications in systemic lupus erythematosus: a meta-analysis of infectious pneumonia and interstitial lung disease

**DOI:** 10.3389/fimmu.2026.1758316

**Published:** 2026-03-18

**Authors:** Ze Yang, Yanzuo Wu, Zexuan Wu, Shuo Huang, Yongsheng Fan, Jie Bao

**Affiliations:** 1 The First School of Clinical Medicine, Zhejiang Chinese Medical University, Hangzhou, Zhejiang, China; 2 School of Basic Medical Sciences, Zhejiang Chinese Medical University, Hangzhou, Zhejiang, China; 3 Department of Rheumatology, The Second Affiliated Hospital of Zhejiang Chinese Medical University, Hangzhou, Zhejiang, China

**Keywords:** infectious pneumonia, interstitial lung disease, meta-analysis, pulmonary complication, risk factors, systemic lupus erythematosus

## Abstract

**Background:**

Pulmonary complications (PC), including infectious pneumonia (IP) and non-infectious interstitial lung disease (ILD), are major contributors of morbidity and mortality in systemic lupus erythematosus (SLE). As they are fundamentally different with respect to their respective etiologies and pathophysiology, we aimed to comprehensively identify and compare their risk factors.

**Methods:**

We conducted a comprehensive literature search in seven electronic databases from database inception to September 2025. Pooled effect sizes were computed using appropriate model-based methods and thoroughly examined for heterogeneity. Our analytical approach advanced an evidence stratification framework that integrated both univariate associations and multivariate-adjusted results, which classified factors into four strata of findings: robust independent risk factors, preliminary independent risk factors, potential risk factors, and protective factors.

**Results:**

In total, 16 studies comprising 6,978 participants yielded fundamentally distinct risk architectures. IP was predominantly driven by immunosuppression and systemic inflammation, with robust independent risk factors including advanced age, pulmonary involvement, high CRP/WBC, immunosuppressant use, and antibacterial drug use. In contrast, ILD was strongly driven by autoimmunity and vascular pathology, with preliminary independent risk factors including Raynaud’s phenomenon, anti-Sm antibody positivity, high IgG and C4 levels. Most strikingly, serum IgG emerged as two strongly associated factors: low levels of serum IgG protected against IP, whereas high levels of serum IgG increased ILD risk.

**Conclusion:**

This is the first study to systematically stratify PC risk factors in SLE, demonstrating their distinct pathogenesis. The hierarchic framework allows a shift from a uniform management mindset to individualized risk evaluation for the respective complications, supportimg targeted prevention and early detection strategies.

**Systematic review registration:**

https://www.crd.york.ac.uk/PROSPERO/view, identifier CRD420251020965.

## 1 Introduction

Systemic lupus erythematosus (SLE) is a prototypical polyautoimmune disease characterised by widespread immune tolerance breakage, the generation of a broad spectrum of autoantibodies, and subsequent inflammatory injury to multiple organs (1). The clinical application of standard treatment algorithms centred around glucocorticoids and immunosuppressive drugs has improved disease control and survival for SLE patients (2). Nevertheless, effective management of SLE continues to be hindered by considerable variabilities in pulmonary complications(PC). These complications are very prevalent, seen in 50-70% of patients and even represent the initial presentation in 4-5% of cases (3). These manifestations also have clinical relevance because they are one of the main causes of hospital admission and poor outcomes in patients (4).

In terms of clinical presentation, these PC can be broadly categorised into two groups: infectious and non-infectious. The immunosuppressive condition induced by therapy, along with underlying immunopathological abnormalities in SLE provide conditions for the occurrence of immunosuppressive-induced infectious pneumonia (IP) (5). IP is one of the most common and acute severe events in SLE, being the foremost cause of direct mortality and the main cause of ICU admission (6, 7). In contrast, the autoimmune attack can be dysregulated and strike the lung structure to cause interstitial lung disease (ILD) (8, 9). ILD presents as a chronic and gradual fibrosing process, leading to a progressive reduction in lung function that exerts an independent impact on long-term functional status and quality of life (10).

The basic pathomechanisms underlying these two complications are per se opposited. IP is usually caused by a weakened immune defense against infections. In contrast, ILD is the consequence of a pro-inflammatory and autoimmune attack on the lung parenchyma. This physiopathological opposition presents a major clinical conundrum: the immunosuppressive drugs that are indispensable to enfeeble the autoimmune attack that causes ILD are also associated with increased risks of severe IP (11, 12). It logically follows that the clinical conditions that predispose patients to PI and ILD are likely to be different, and possibly even inversely correlated. However, a direct comparison to elucidate these two different risk profiles is still lacking in the current literature. Although these two individual entities (infection and ILD) of clinical importance in SLE have been studied individually, a synthesized analysis directly juxtaposing their risk factors is still lacking. A precise understanding of the different risk factors for IP versus ILD is important to move from a generalized management strategy towards a personalized one to better predict and prevent the different complications that are most likely to affect the individual patient.

This study therefore attempts to conduct a systematic review and meta-analysis to explore the independent risk factors, both univariate and multivariate, associated with the occurrence of IP and non-infectious ILD in SLE patients, offering a clearer and more evidence-based understanding to help clinicians in the risk stratification, prevention, and management of the two most consequential and pathophysiologically distinct PC in SLE.

## 2 Materials and methods

The study design and reporting of this systematic review and meta-analysis followed closely the Preferred Reporting Items for Systematic Reviews and Meta-Analyses (PRISMA) guidelines (13). Furthermore, the study protocol has been prospectively registered on the International Prospective Register of Systematic Reviews (PROSPERO, registration number **) to standardize the research process, prevent duplication, and minimize selective reporting bias. Patients and the public will be involved in this research for conducting and reporting.

### 2.1 Inclusion and exclusion criteria

Inclusion Criteria: ① Patients meeting the 1997 American College of Rheumatology (ACR) diagnostic criteria for SLE; ② Patients aged over 18 years; ③ Studies reporting risk factors for IP/ILD with effect measures, such as odds ratios(OR) and 95% confidence intervals(CI); ④ Observational studies (including cohort, case-control, and cross-sectional designs); ⑤ Publications in both Chinese and English.

Exclusion Criteria: ① Patients with overlapping connective tissue diseases (e.g., rheumatoid arthritis, systemic sclerosis) or confirmed active malignancies; ② Studies lacking an appropriate control group; ③ Duplicate, incomplete, or low-quality studies; ④ Basic research, conference abstracts, reviews, books, etc.; ⑤ Studies with inaccessible full text or non-extractable data.

### 2.2 Literature search strategy

We conducted a systematic search of PubMed, EMBASE, Web of Science, China National Knowledge Infrastructure (CNKI), Wanfang Database, Chinese Biomedical Literature Database (CBM), and VIP, covering literature published from database inception to September 2025. Search terms included “Systemic lupus erythematosus”, “Infectious pneumonia”, “Interstitial lung disease” and “risk factors” using a combination of mesh terms and free-text terms with Boolean operators. We also manually screened the reference lists of relevant reviews to capture any potentially missed studies. Additionally, grey literature meeting the inclusion criteria was searched in OpenGrey, ClinicalTrials.gov, and the WHO Clinical Trial Registration Center. For example, the search strategy used in PubMed is detailed in **Supplementary Table 1**.

### 2.3 Literature screening and data extraction

Two researchers (** and **) independently screened titles and abstracts and evaluated full texts; discrepancies were resolved through discussion or adjudication by a third party (**). Extracted data included the first author, publication year, study design, age, gender(female%), sample size, SLE duration, risk factors, the type of PC and NOS scores. Concerning the risk factor of using antibacterial drugs, it was operationalized using the original studies as the reported use of systemic antibacterial drugs before the diagnosis of the episode of pneumonia. The quality of the studies was assessed using the Newcastle–Ottawa Scale (NOS), with each item assigned 1 point, for a maximum score of 9. Scores of 0–4 were considered low quality, 5–6 moderate quality, and 7–9 high quality (14).

### 2.4 Statistical analysis

Given the fundamental etiological and pathophysiological distinctions between IP and non-infectious ILD, this study did not pool these outcomes to calculate an overall effect size. All risk factors were subjected to separate subgroup meta-analyses specifically for each of the two PC. This study employed RevMan 5.3 and Stata 15.0 for data analysis. For dichotomous variables, ORs were calculated, while mean differences (MDs) or standardized mean differences (SMDs) were used for continuous variables. Heterogeneity was evaluated using the Q test and I² statistic; if I² was ≥50% or the Q test yielded a p-value <0.10, a random-effects model (REM) was adopted, otherwise a fixed-effects model (FEM) was applied. To understand the possible origins of the heterogeneity, the subgroup analyses stratified by study design and sample size were conducted, and sensitivity analyses were conducted sequentially after excluding studies and switching between effect models to test the stability of the results. Publication bias will be assessed by funnel plots (for visual inspection when ≥10 studies are available for the corresponding summary) and Egger’s linear regression test. If the result of Egger’s test is statistically significant (p<0.05), the existence of publication bias is suggested. All CI and p-values were reported, and p<0.05 was considered statistically significant.

For the synthesis of multivariate analysis results, we have used a synthesized, narrative summary approach when the number of independent studies in a given risk factor is insufficient to conduct a traditional meta-analysis.

### 2.5 Evidence stratification framework

Given the need to rigorously interpret the strength and clinical importance of the detected risk factors, we constructed a hierarchical evidence stratification system in which factors were categorized based on the following definitions: ①Robust independent risk factors: A factor that exhibits a significant independent association (p < 0.05) in a multivariate meta-analysis of two or more studies. ②Preliminary independent risk factors: A factor that exhibits a significant independent association (p < 0.05) in a multivariate analysis but based on results from one study. ③Potential risk factors: A factor that is significant (p < 0.05) in univariate analysis only, potentially due to another variable confounding its association. ④Protective factors: A factor that exhibits a significant inverse association (p < 0.05) with the complication in either a univariate or multivariate analysis.

This stratification may enable more meaningful discussion of our results with regard to the most robust evidence-level factors versus those that need further confirmation.

## 3 Results

### 3.1 Literature search results

**Figure 1:**
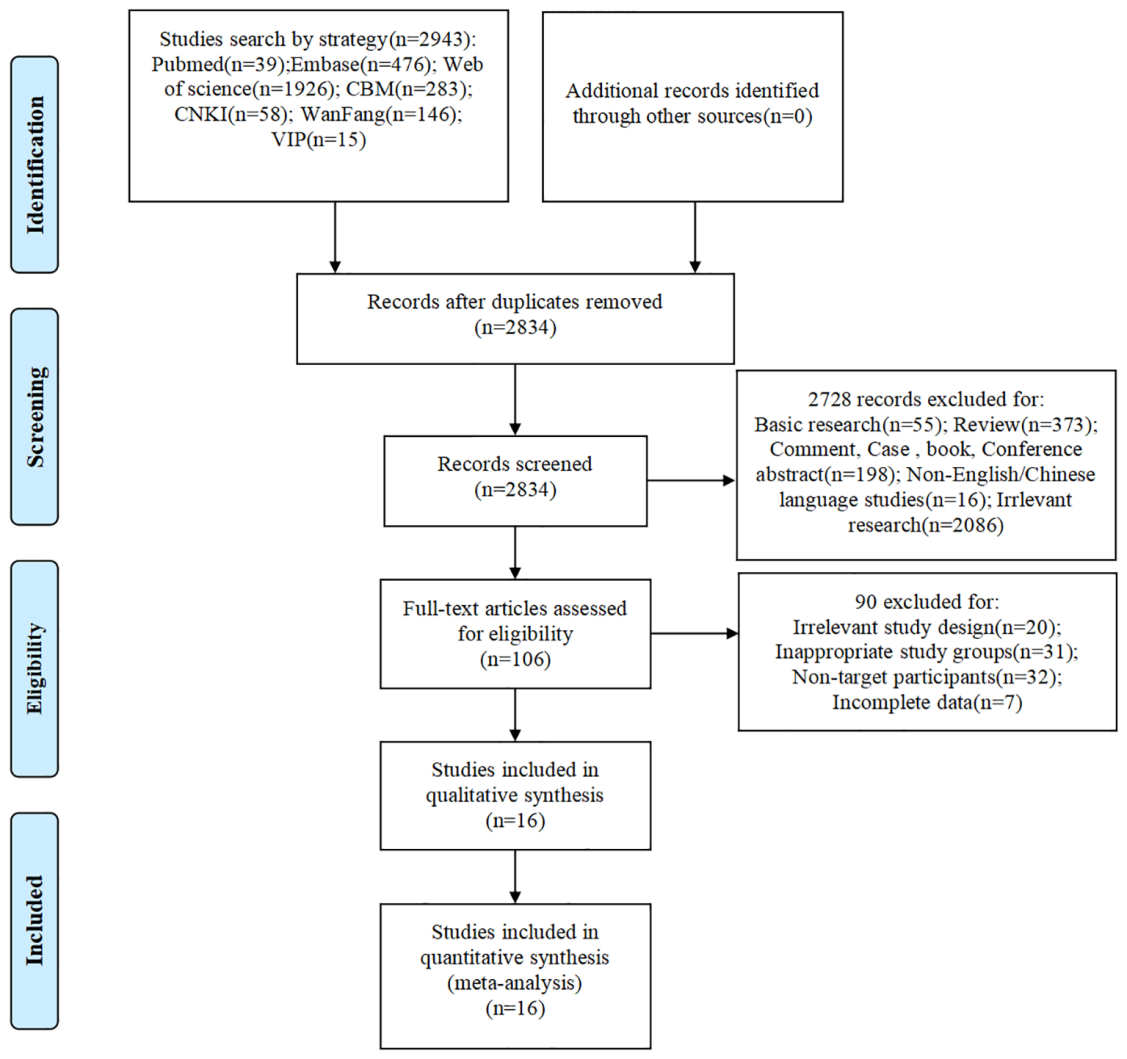
Literature screening of risk factors for PC in SLE patients PRISMA flow chart.

A total of 2,943 records were found through search. No additional records were found through other sources. After removal of duplicates, 2,834 records remained for initial screening. During initial screening, 2,728 records were excluded for not fulfilling inclusion criteria, resulting in 106 articles for full text assessment. Of these 90 articles were excluded in full text review for having irrelevant study design (n=20), inappropriate study groups (n=31), non-target participants (n=32) and insufficient data (n=7). Finally, 16 studies (15–30) were retained for qualitative synthesis which were all included in quantitative synthesis as well (**Figure 1**).

### 3.2 Characteristics and quality assessment of included studies

Among the 16 included studies, 11 reported on IP and 5 on ILD. The studies consisted of 9 case-control studies and 7 cohort studies published between 2014 and 2023. The number of cases varied between 58 and 2,288, totalling to 6,978 subjects in all. With regard to risk factor analyses, most of the studies reported univariate results but some studies further analyzed the same using multivariate analysis. Quality assessment through the Newcastle–Ottawa Scale (NOS) showed scores between 7 and 9 points, indicating that the studies were of high quality overall. The detailed characteristics and quality assessment results are summarized in **Table 1**.

**Table 1:** Characteristics and quality assessment of the 16 included studies.

Study	Study design	Age (years)	Gender(female%)	Sample (PC/without PC)	SLE course	Risk factors(univariate data)	Risk factors(multivariate data)	The type of PC	NOS scores
Chen 2018	Case-control study	ILD: 35.45 ± 13.40 Non-ILD: 37.90 ± 16.70	83.8	40/40	–	①②⑥⑦⑧⑩⑫⑬㉒㉘㉙㉚㉛㉜㉟	⑬㉙㉜	ILD	7
Chen ZX 2018	Cohort study	ILD: 44.43 ± 15.22 Non-ILD: 33.58 ± 14.19	86.9	138/2150	ILD: 39.05 ± 69.46 Non-ILD: 31.80 ± 58.75	①②③④⑥⑦⑧⑨⑩⑪⑫⑬⑭⑮⑱⑲㉑㉒㉓㉔㉖㉗㉘㉙㉚㉛㉜㉝㉞㉟㊱	①㉗㊱	ILD	8
Fan 2014	Case-control study	IP: 45.0 ± 19.0 Non-IP: 37.0 ± 22.0	82.8	22/36	–	①②⑤㉒㉓㊷㊺㊻㊼	⑰㊺㊻	IP	7
Guo 2020	Cohort study	–	91.0	27/73	–	①②③④⑪⑯㉖㊷㊻㊼	③⑯㉖㊷㊻㊼	IP	7
Li 2018	Case-control study	IP: 40.78 ± 15.81 Non-IP: 36.12 ± 11.67	91.8	85/85	–	①②③④⑯⑰⑱⑲⑳㉑㉒㉓㉔㉕㉖㉗㉘㊱㊲㊳㊵㊶㊷㊺㊻㊼㊽	–	IP	7
Liao 2021	Case-control study	ILD: 47.38 ± 11.58 Non-ILD: 42.15 ± 13.40	95.6	34/34	ILD: 72.00(0.60, 480.00) Non-ILD: 29.42(1.23, 350.56) months	①②④⑥⑦⑧⑨⑩⑫⑬⑱⑲⑳㉑㉒㉓㉔㉖㉗㉘㉙㉚㉛㉜㊱㊲㊳㊺㊼㊽	④㉒㉖㉘㊳	ILD	7
Liu 2023	Cohort study	IP: 49.00 ± 16.90 Non-IP: 38.80 ± 13.50	95.2	62/188	IP: 21.00(4.00, 42.00) Non-IP:15.00 (4.00, 36.00) months	①②④⑱⑲㉓㉔㊴㊹㊺	①⑱⑲	IP	8
Qiu 2021	Cohort study	IP: 36.00 ± 24.00 Non-IP: 30.00 + 20.00	82.9	397/1637	IP: 3.00(48.00) Non-IP:2.00(36.00) months	①②③④⑥⑦⑧⑨⑩⑪⑫⑬⑭⑲㉒㉓㉔㉕㉖㉗㉘㉙㉚㉛㉜㉝㉞㉟㊱㊲㊳㊷㊸㊹	①③⑥⑧⑫㉓㊱㊷	IP	7
Rúa-Figueroa 2014	Case-control study	13-84	92.0	30/196	12.70 ± 8.40 months	⑩⑮⑯⑰㊺㊼㊽㊾	㊾	IP	9
Tao 2014	Cohort study	34.61 ± 12.14	77.8	51/116	–	②⑯⑰⑲⑳㉖㉘㉙㊺㊼	⑰㊺㊼	IP	7
Wang 2020	Case-control study	IP: 49.77 ± 9.85 Non-IP: 40.33 ± 12.68	87.7	13/52	IP: 36 (5.50, 120.00) Non-IP: 36 (4.00, 120.00) months	①②④⑰⑱⑲⑳㉒㉓㉔㉕㉖㉗㉘㉚㉛㉜㊴㊶㊷	㉓	IP	7
Wang 2024	Cohort study	ILD: 51.00 (43.50, 61.00) Non-ILD: 42.00 (30.00, 51.00)	94.8	58/343	ILD: 7.50 (2.00, 11.00) Non-ILD: 6.00 (1.00, 10.00) years	①②③④⑦⑧⑨⑩⑫⑬⑭㉒㉓㉔㉖㉗㉘㉙㉚㉛㉜㉝㉞㉟㊱㊲㊳㊴㊵㊷㊸㊹	㉒㊲	ILD	6
Wu 2021	Cohort study	IP: 44.9 ± 15.2 Non-IP: 39.5 ± 14.6	89.2	168/527	–	①②③⑥⑦⑧⑨⑩⑫⑬⑭⑮ ⑲⑳㉓㉔㉖㉗㉘㉙㉚㉛㉜㉝㉞㉟㊱㊲㊳㊴㊵㊶㊷㊸㊹㊺㊼㊽	①㊴㊷㊸	IP	7
Xia 2021	Case-control study	ILD: 50.17 ± 11.86 Non-ILD: 51.90 ± 12.75	93.3	30/30	–	①②④⑥⑦⑨⑩⑫⑬⑱⑲⑳㉑㉓㉔㉖㉗㉘㉙㉚㉛㉜㊱㊲㊳㊴㊷㊸㊹	㊴	ILD	7
Zhou 2015	Case-control study	IP:34.5 ± 15.2 Non-IP:31.70 ± 11.90	82.5	80/80	33.0 ± 47.50	⑤㉓㉔㉖㊴㊷㊺	–	IP	8
Zhou 2016	Case-control study	IP:36.99 ± 15.73 Non-IP:32.09 ± 9.12	94.9	78/78	IP: 74.85 ± 102.32 Non-IP: 95.60 ± 88.08	①④⑯⑱⑲⑳㉑㉒㉓㉔㉖㉗㉘㊴㊵㊷㊺㊼	–	IP	7

①Age ②Gender ③Hospitalization duration ④Disease course ⑤Temperature ⑥Fever ⑦Rash ⑧Photosensitivity ⑨Alopecia ⑩Cutaneous and mucosal ulcers⑪Serositis ⑫Arthritis ⑬Raynaud's phenomenon ⑭Xerostomia and dry eyes ⑮Vasculitis ⑯Involvement of major organs(≥2) ⑰Pulmonary involvement ⑱Cardiac involvement ⑲Renal involvement ⑳Neurological involvement ㉑Hematological involvement ㉒SLEDAI score ㉓CRP ㉔ESR ㉕PCT ㉖C3 ㉗C4 ㉘Anti-dsDNA antibody ㉙Anti-SM antibody ㉚Anti-SSA antibody ㉛Anti-SSB antibody ㉜Anti-U1RNP antibody ㉝Anti-Jo-1 antibody ㉞Anti-Scl-70 antibody ㉟Anti-histone antibody ㊱IgG ㊲IgA ㊳IgM ㊴Albumin ㊵Globulin ㊶D-dimer ㊷WBC ㊸Hb ㊹PLT ㊺GC ㊻Antibacterial drug ㊼Immunosuppressants ㊽CTX ㊾FCGR2A HH genotype.

NOS, the Newcastle-Ottawa Scale; ILD, Interstitial lung disease; IP, Infectious pneumonia; SLE, Systemic lupus erythematosus.

### 3.3 Meta-analysis results

**Figure 2:**
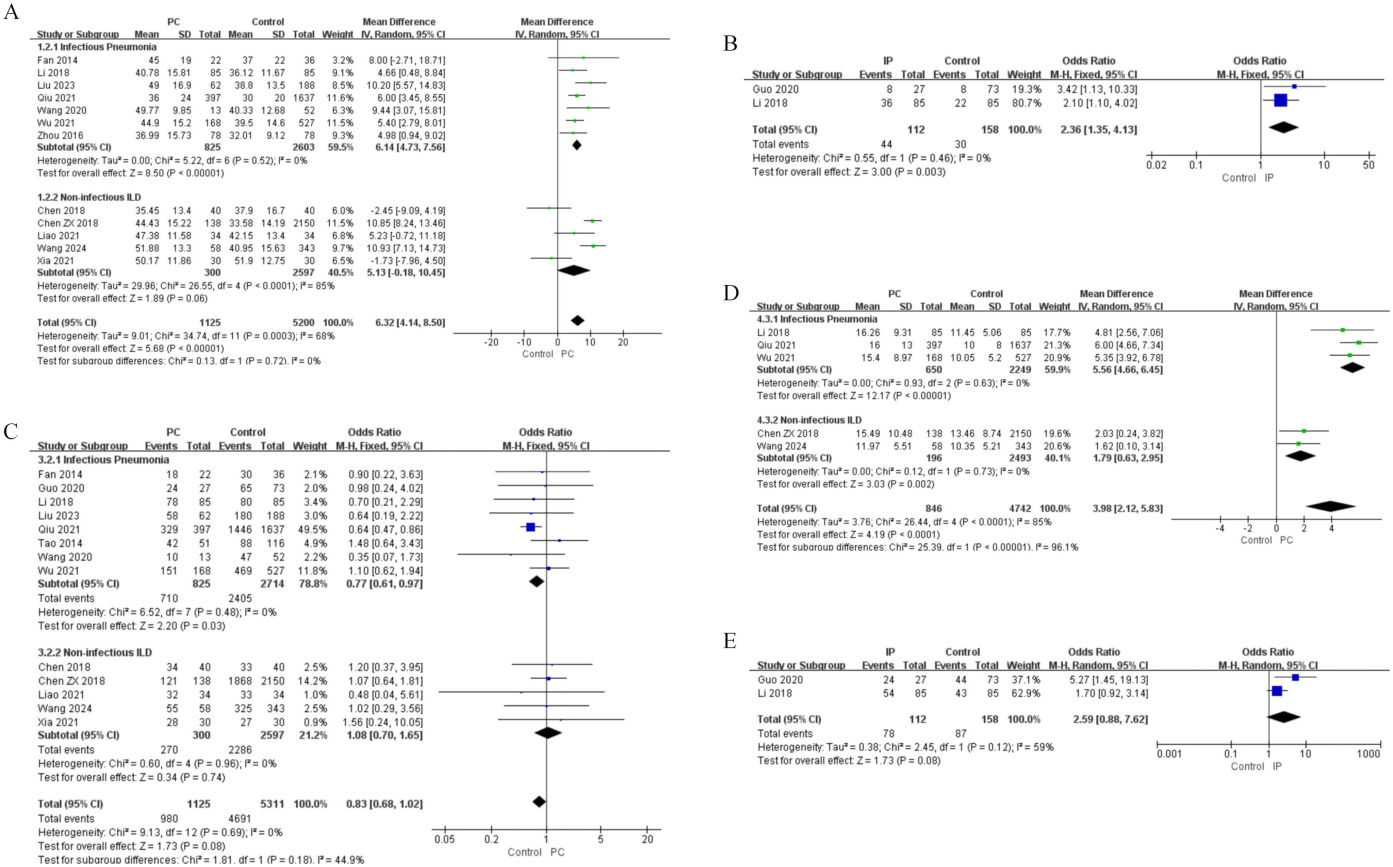
Forest plots of univariate analysis for demographic characteristics in SLE patients with IP and non-infectious ILD. **(A)** Age (continuous variable) **(B)** Age (dichotomous variable) **(C)** Gender **(D)** Disease Course(continuous variable) **(E)** Disease Course (dichotomous variable).

**Figure 3:**
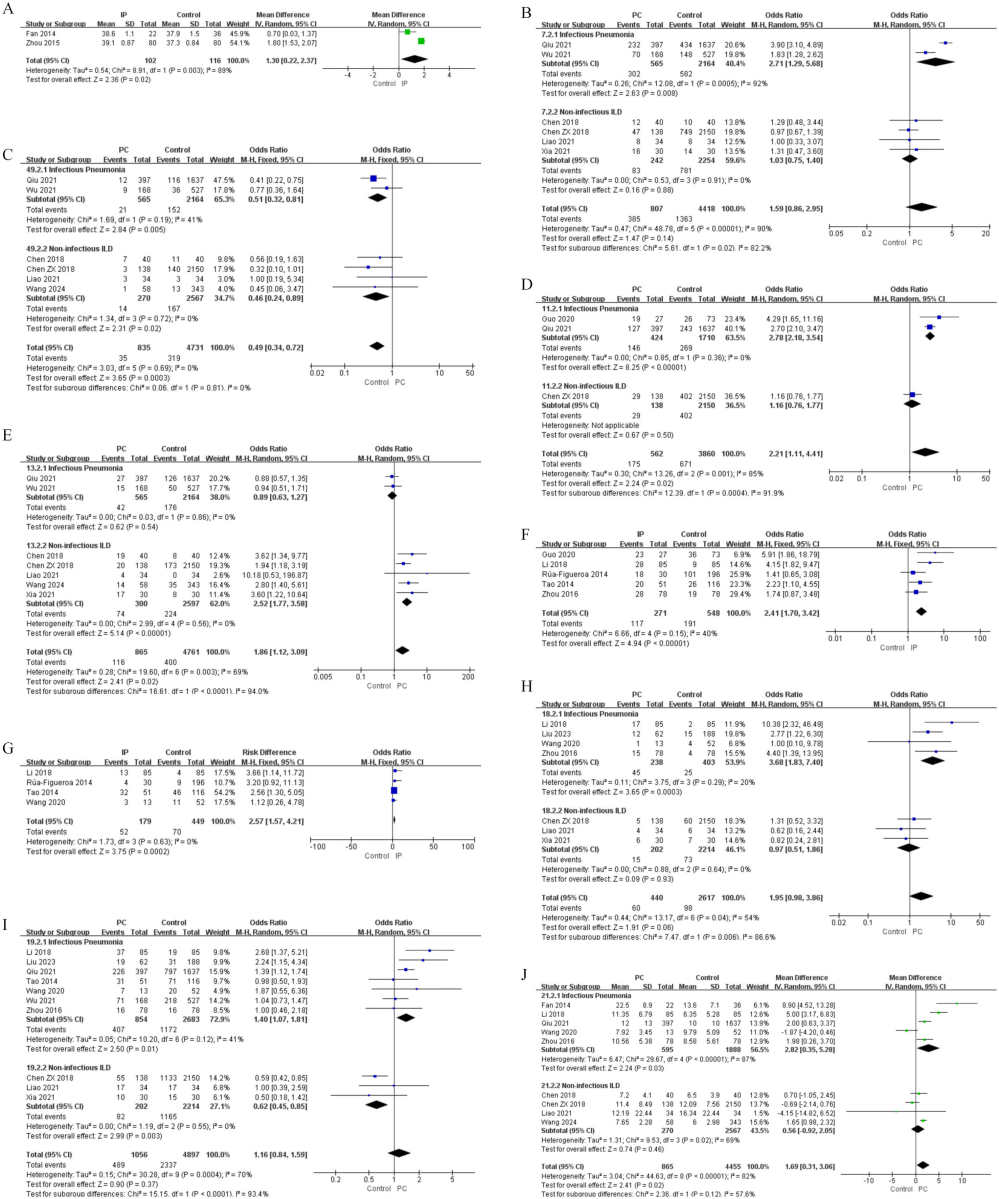
Forest plots of univariate analysis for clinical features in SLE patients with IP and non-infectious ILD. **(A)** Temperature **(B)** Fever **(C)** Photosensitivity **(D)** Serositis **(E)** Raynaud’s Phenomenon **(F)** Involvement of major organs(≥2) **(G)** Pulmonary Involvement **(H)** Cardiac Involvement **(I)** Renal Involvement **(J)** SLEDAI Score.

**Figure 4:**
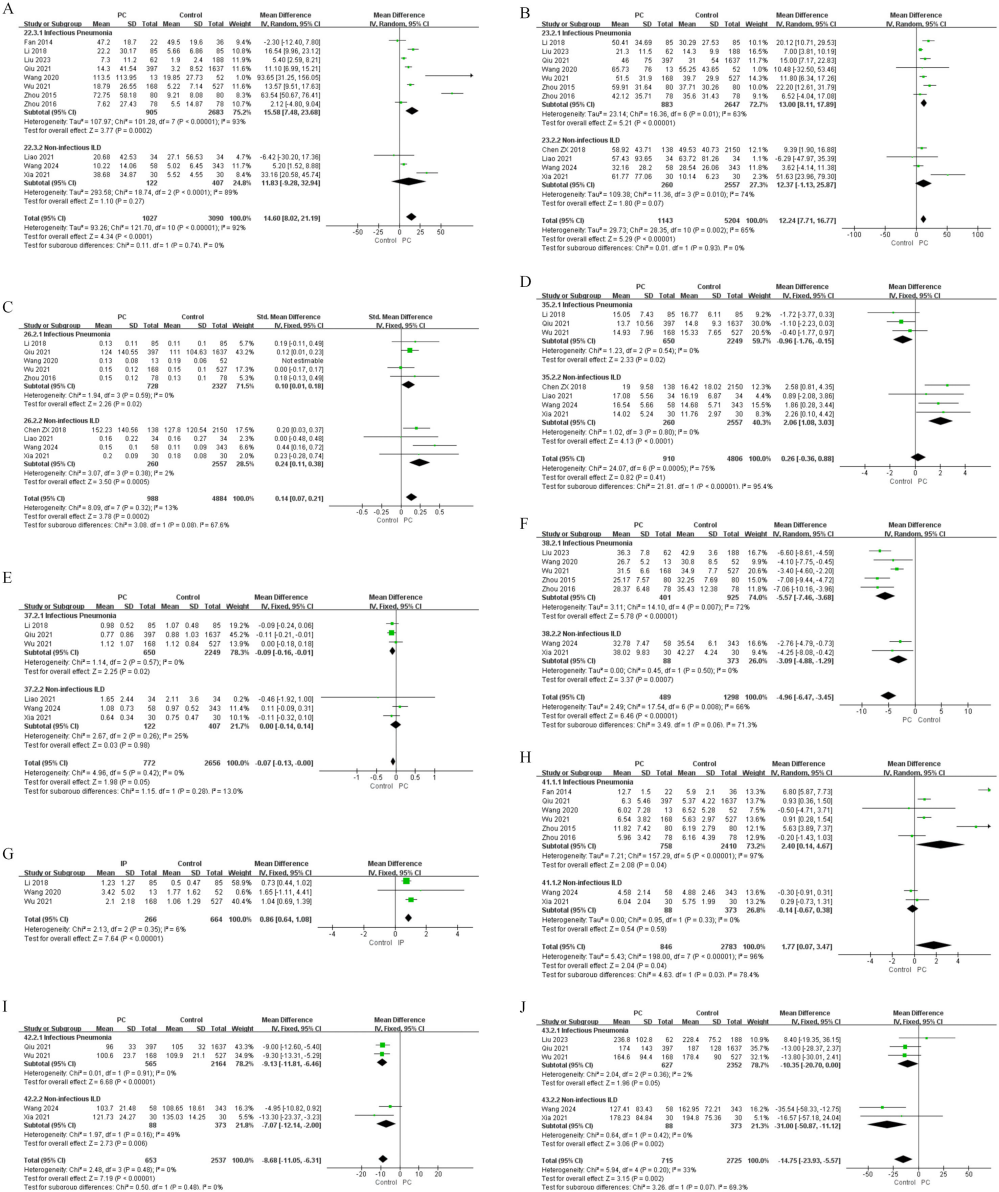
Forest plots of univariate analysis for laboratory findings in SLE patients with IP and non-infectious ILD. **(A)** CRP **(B)** ESR **(C)** C4 **(D)** IgG **(E)** IgM **(F)** Albumin **(G)** D-dimer **(H)** WBC **(I)** Hb **(J)** PLT.

**Figure 5:**
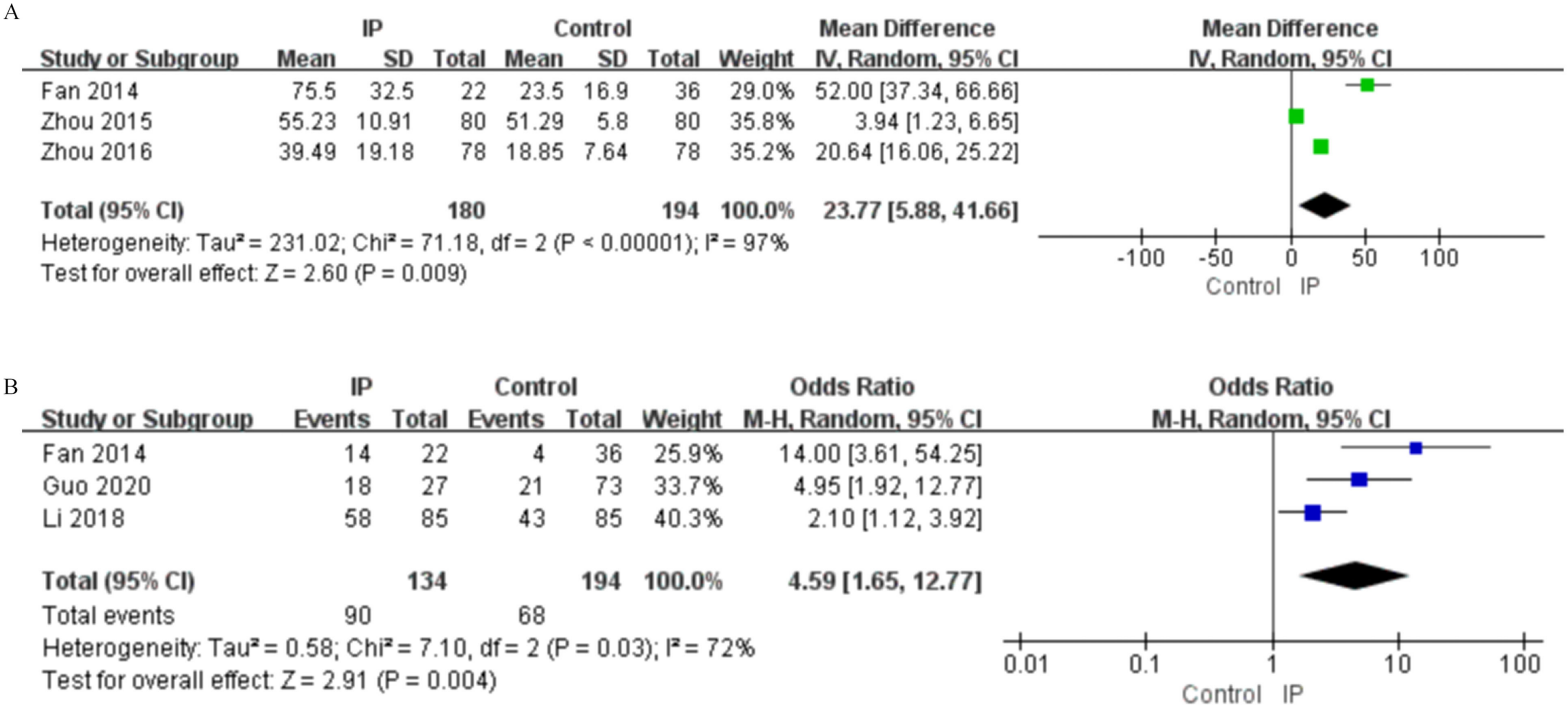
Forest plots of univariate analysis for medication use in SLE patients with IP and non-infectious ILD. **(A)** Glucocorticoids (Continuous Variable) **(B)** Antibacterial Drug Use.

**Figure 6:**
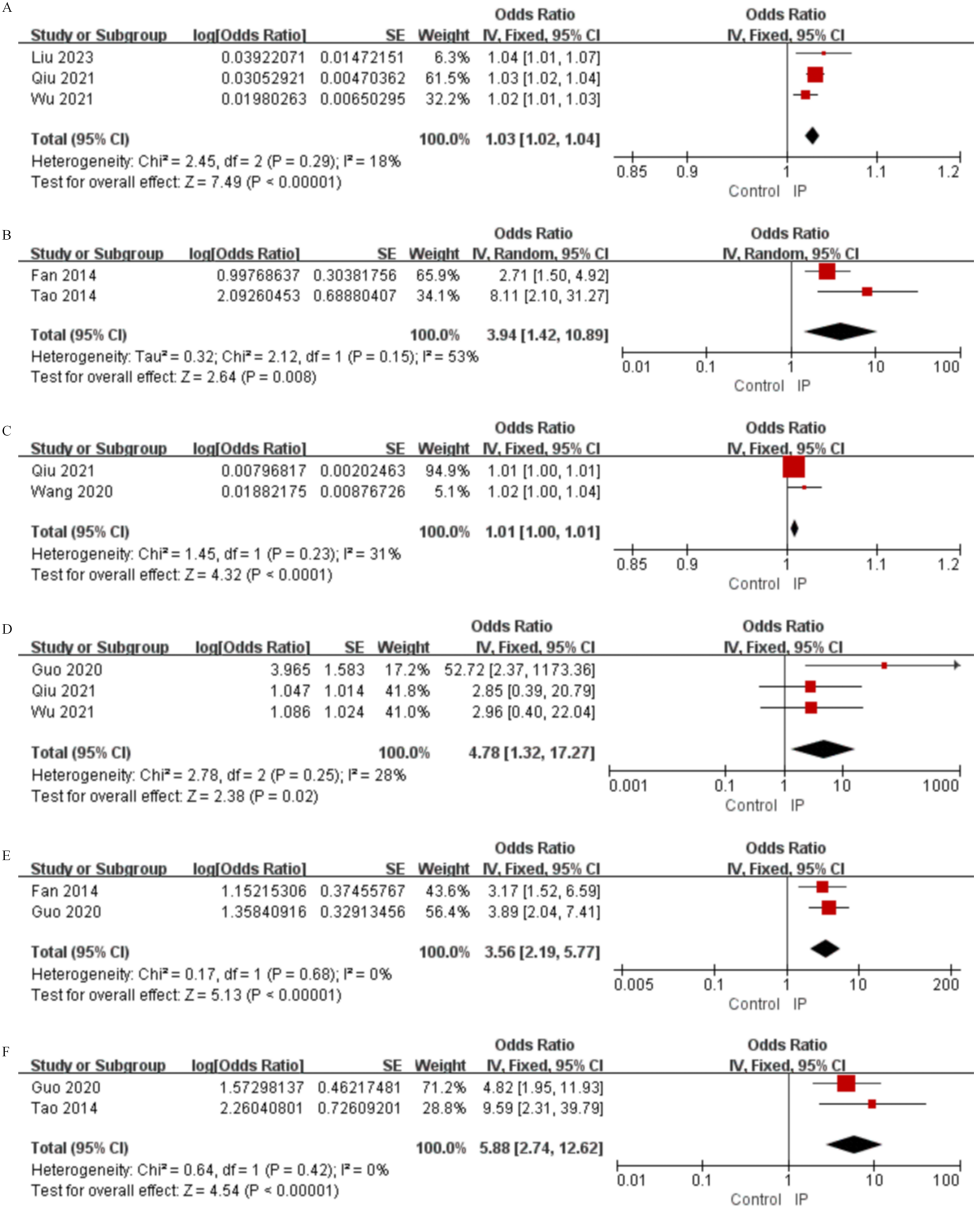
Forest plots of multivariate analysis for IP in SLE patients. **(A)** Age **(B)** Pulmonary involvement **(C)** CRP **(D)** WBC **(E)** Antibacterial drugs **(F)** Immunosuppressants.

Risk factors were classified into five groups: demographic characteristics, clinical features, laboratory findings, medication use and immunogenetic factors. The results of univariate and multivariate analyses are shown in **Figures 2**–**6**, **Tables 2**–**5**.

**Table 2:** Meta-analysis results of univariate analysis for related factors of IP in SLE patients.

Risk factors	Study number	Sample	I^2^ value	Effect model	Effect size	95%CI	*P* value
Demographic characteristics							
Age^1^	7	3428	0	FEM	MD=6.14	4.73, 7.56	**<0.00001**
Age^2^	2	270	0	FEM	OR=2.63	1.35, 4.13	**0.003**
Gender	8	3539	0	FEM	OR=0.77	0.61, 0.97	**0.030**
Hospitalization duration^1^	3	2899	0	FEM	MD=5.56	4.66, 6.45	**<0.00001**
Hospitalization duration^2^	2	270	0	FEM	OR=3.31	1.56, 7.04	**0.002**
Disease course^1^	4	2505	0	FEM	SMD=0.02	-0.08, 0.11	0.720
Disease course^2^	2	270	59	REM	OR=2.59	0.88, 7.62	0.080
Clinical features							
Temperature	2	218	89	REM	MD=1.30	0.22, 2.37	**0.020**
Fever	2	2729	92	REM	OR=2.71	1.29, 5.68	**0.008**
Rash	2	2729	40	FEM	OR=0.83	0.68, 1.02	0.070
Photosensitivity	2	2729	41	FEM	OR=0.51	0.32, 0.81	0.050
Alopecia	2	471	0	FEM	OR=0.96	0.75, 1.23	0.720
Cutaneous and mucosal ulcers	3	2955	75	REM	OR=1.78	0.81, 3.90	0.150
Serositis	2	2134	0	FEM	OR=2.79	2.19, 3.56	**<0.00001**
Arthritis	2	2729	0	FEM	OR=0.87	0.70, 1.09	0.240
Raynaud's phenomenon	2	2729	0	FEM	OR=0.90	0.66, 1.23	0.520
Xerostomia and dry eyes	2	2729	0	FEM	OR=0.90	0.66, 1.23	0.520
Vasculitis	2	921	85	REM	OR=1.15	0.13, 10.32	0.900
Involvement of major organs(≥2)	5	819	40	FEM	OR=2.41	1.70, 3.42	**<0.00001**
Pulmonary involvement	4	628	0	FEM	OR=2.57	1.57, 4.21	**0.0002**
Cardiac involvement	4	641	20	FEM	OR=3.97	2.26, 6.98	**<0.00001**
Renal involvement	7	3537	41	FEM	OR=1.36	1.15, 1.60	**0.0002**
Neurological involvement	5	1253	0	FEM	OR=1.40	0.87, 2.27	0.160
Hematological involvement	2	326	0	FEM	OR=1.06	0.67, 1.66	0.820
SLEDAI score	5	2483	87	REM	MD=2.82	0.35, 5.28	**0.030**
Laboratory findings							
CRP	8	3588	93	REM	MD=15.58	7.18, 23.68	**0.0002**
ESR	7	3530	63	REM	MD=13.00	8.11, 17.89	**<0.00001**
PCT	3	2269	77	REM	MD=1.18	-0.21, 2.58	0.100
C3^1^	6	3280	79	REM	SMD=-0.22	-0.44, 0.00	0.050
C3^2^	3	437	57	REM	OR=0.73	0.36, 1.50	0.390
C4	4	3055	0	FEM	SMD=0.10	0.01, 0.18	0.020
Anti-dsDNA antibody^1^	2	2204	0	FEM	MD=-14.85	-33.32, 3.62	0.120
Anti-dsDNA antibody^2^	4	1083	0	FEM	OR=0.89	0.67, 1.20	0.450
Anti-SM antibody	3	2896	0	FEM	OR=0.86	0.69, 1.05	0.140
Anti-SSA antibody	3	2794	0	FEM	OR=1.00	0.83, 1.20	0.970
Anti-SSB antibody	3	2794	0	FEM	OR=1.02	0.80, 1.30	0.89
Anti-U1RNP antibody	3	2794	0	FEM	OR=0.93	0.76, 1.12	0.430
Anti-Jo-1 antibody	2	2729	27	FEM	OR=1.61	0.70, 3.73	0.260
Anti-Scl-70 antibody	2	2729	60	REM	OR=0.47	0.09, 2.55	0.380
Anti-histone antibody	2	2729	54	REM	OR=1.41	0.63, 3.20	0.410
IgG	3	2899	0	FEM	MD=-0.96	-1.76, -0.15	**0.020**
IgA	3	2899	0	FEM	MD=-0.04	-0.19, 0.11	0.580
IgM	3	2899	0	FEM	MD=-0.09	-0.16, -0.01	**0.020**
Albumin	5	1326	72	REM	MD=-5.57	-7.46, -3.68	**<0.00001**
Globulin	3	1021	0	FEM	MD=0.30	-0.93, 1.53	0.640
D-dimer	3	930	6	FEM	MD=1.04	0.69, 1.39	**<0.00001**
WBC^1^	6	3168	97	REM	MD=2.40	0.14, 4.67	**0.040**
WBC^2^	2	270	71	REM	OR=0.58	0.19, 1.77	0.340
Hb	2	2729	0	FEM	MD=-9.13	-11.81, -6.46	**<0.00001**
PLT	3	2979	2	FEM	MD=-10.35	-20.70, 0.00	0.050
**Medication use**				REM	OR=1.98		
GC^1^	3	374	97	REM	MD=23.77	5.88, 41.66	**0.009**
GC^2^	5	1508	76	REM	OR=1.25	0.96, 1.62	0.090
Antibacterial drug	3	328	72	REM	OR=4.59	1.65, 12.77	**0.004**
Immunosuppressants	7	1572	88	REM	OR=1.87	0.83, 4.20	0.130
CTX	2	396	0	FEM	OR=1.60	0.87, 2.97	0.130
Immunogenetic Factors							
FCGR2A HH genotype	1	158	–	–	OR=2.9	1.09, 7.68	**0.030**

^1^Continuous variable, ^2^Dichotomous variable.

CI, Confidence Interval; FEM, Fixed-Effect Model; OR, Odds Ratio; MD, Mean Difference; REM, Random-Effects Model; SMD, Standardized Mean Difference.The bold values indicate statistical significance with p < 0.05.

**Table 3:** Meta-analysis results of univariate analysis for related factors of non-infectious ILD in SLE patients.

Risk factors	Study number	Sample	I^2^ value	Effect model	Effect size	95%CI	*P* value
Demographic characteristics							
Age^1^	5	2897	85	REM	MD=5.13	-0.18, 10.45	0.060
Gender	5	2897	0	FEM	OR=1.08	0.70, 1.65	0.740
Hospitalization duration^1^	2	2689	0	FEM	MD=1.79	0.63, 2.95	**0.002**
Disease course^1^	4	2817	18	FEM	SMD=0.10	-0.03, 0.24	0.130
Clinical features							
Fever	4	2496	0	FEM	OR=1.02	0.75, 1.40	0.880
Rash	5	2897	53	REM	OR=0.83	0.53, 1.31	0.430
Photosensitivity	4	2837	0	REM	OR=0.46	0.24, 0.89	**0.020**
Alopecia	4	2817	45	REM	OR=0.86	0.60, 1.23	0.420
Cutaneous and mucosal ulcers	5	2897	16	FEM	OR=1.01	0.68, 1.50	0.950
Serositis	1	2288	–	–	OR=1.16	0.76, 1.77	0.500
Arthritis	5	2897	0	FEM	OR=1.16	0.89, 1.52	0.260
Raynaud's phenomenon	5	2897	0	FEM	OR=2.62	1.86, 3.69	**<0.00001**
Xerostomia and dry eyes	2	2689	0	FEM	OR=1.48	0.93, 2.34	0.100
Vasculitis	1	2288	–	–	OR=1.61	0.90, 2.87	0.110
Cardiac involvement	3	2416	0	FEM	OR=0.95	0.49, 1.86	0.890
Renal involvement	3	2416	0	FEM	OR=0.62	0.45, 0.85	**0.003**
Neurological involvement	3	2416	0	FEM	OR=1.19	0.46, 3.08	0.720
Hematological involvement	3	2416	28	FEM	OR=0.88	0.64, 1.20	0.420
SLEDAI score	4	2837	69	FEM	MD=0.56	-0.92, 2.05	0.460
Laboratory findings							
CRP	4	2817	86	REM	MD=9.07	-0.99, 19.12	0.080
ESR	4	2817	74	REM	MD=12.37	-1.13, 25.87	0.070
C3^1^	4	2817	12	FEM	SMD=0.13	-0.01, 0.26	0.070
C4	4	2817	2	FEM	SMD=0.24	0.11, 0.38	**0.0005**
Anti-dsDNA antibody^1^	1	68	–	–	MD=-11.10	-137.39, 115.19	0.860
Anti-dsDNA antibody^2^	4	2829	55	REM	OR=0.76	0.46, 1.27	0.300
Anti-SM antibody	5	2897	39	FEM	OR=1.12	0.84, 1.49	0.460
Anti-SSA antibody	5	2897	16	FEM	OR=0.97	0.75, 1.26	0.820
Anti-SSB antibody	5	2897	35	FEM	OR=0.91	0.65, 1.28	0.600
Anti-U1RNP antibody	5	2897	68	REM	OR=1.62	0.93, 2.84	0.090
Anti-Jo-1 antibody	2	2689	6	FEM	OR=2.48	0.62, 9.90	0.200
Anti-Scl-70 antibody	2	2689	0	FEM	OR=1.54	0.60, 3.95	0.370
Anti-histone antibody	3	2769	0	FEM	OR=1.03	0.68, 1.56	0.880
IgG	4	2817	0	FEM	MD=2.06	1.08, 3.03	**<0.0001**
IgA	3	529	91	REM	MD=0.24	-0.64, 1.12	0.600
IgM	3	529	25	FEM	MD=0.00	-0.14, 0.14	0.980
Albumin	2	461	0	FEM	MD=--3.09	-4.88, -1.29	**0.0007**
Globulin	1	401	–	–	MD=2.24	-0.34, 4.82	0.090
WBC^1^	2	461	0	FEM	MD=-0.14	-0.67, 0.38	0.590
Hb	2	461	49	FEM	MD=-7.07	-12.14, -2.00	**0.006**
PLT	2	461	0	FEM	MD=-31.00	-50.87, -11.12	**0.002**
Medication use							
GC^2^	1	68	–	–	OR=0.48	0.14, 1.62	0.240
Immunosuppressants	1	68	–	–	OR=0.10	0.01, 1.90	0.120
CTX	1	68	–	–	OR=0.62	0.24, 1.62	0.330

^1^Continuous variable, ^2^Dichotomous variable.

CI, Confidence Interval; FEM, Fixed-Effect Model; OR, Odds Ratio; MD, Mean Difference; REM, Random-Effects Model; SMD, Standardized Mean Difference.The bold values indicate statistical significance with p < 0.05.

**Table 4:** Meta-analysis results of multivariate analysis for related factors for IP in SLE patients.

Risk factors	Study number	I^2^ value	Effect model	OR	95%CI	*P* value
Demographic features						
Age	3	18	FEM	1.03	1.02, 1.04	**<0.00001**
Hospitalization duration	2	90	REM	2.18	0.46, 10.32	0.330
Clinical features						
Fever	1	–	–	3.083	2.137, 4.104	**<0.00001**
Photosensitivity	1	–	–	0.458	0.224, 0.939	**0.033**
Arthritis	1	–	–	0.691	0.508, 0.939	**0.018**
Involvement of major organs(≥2)	1	–	–	4.194	1.294, 5.757	**<0.00001**
Cardiac involvement	1	–	–	1.12	0.33, 3.80	0.860
Renal involvement	1	–	–	0.59	0.22, 1.58	0.290
Pulmonary involvement	2	53	REM	3.94	1.42, 10.89	**0.008**
Laboratory findings						
CRP	2	31	FEM	1.01	1.00, 1.01	**<0.0001**
C3	1	–	–	1.39	1.10, 2.85	**0.010**
IgG	1	–	–	0.98	0.96, 1.00	**0.010**
Albumin	1	–	–	2.15	1.41, 3.27	**<0.0001**
WBC	3	28	FEM	4.78	1.32, 17.27	**0.020**
Hb	1	–	–	1.711	1.046, 2.800	**0.030**
Medication use						
GC	2	76	REM	3.27	0.70, 15.28	0.130
Antibacterial drugs	2	0	FEM	3.56	2.19, 5.77	**<0.00001**
Immunosuppressants	2	0	FEM	5.88	2.74, 12.62	**<0.00001**
Immunogenetic Factors						
FCGR2A HH genotype	1	–	–	3.08	1.25, 7.76	**0.017**

CI, Confidence Interval; FEM, Fixed-Effect Model; OR, Odds Ratio; REM, Random-Effects Model.The bold values indicate statistical significance with p < 0.05.

**Table 5:** Meta-analysis results of multivariate analysis for related factors for non-infectious ILD in SLE patients.

Risk factors	Study number	I^2^ value	Effect model	OR	95%CI	*P* value
Demographic features						
Age	1	–	–	1.05	1.04, 1.07	**<0.00001**
Disease course	1	–	–	0.98	0.99, 1.00	0.390
Clinical features						
Raynaud's phenomenon	1	–	–	3.62	1.34, 9.77	**0.010**
SLEDAI score	2	92	REM	0.98	0.67, 1.43	0.910
Laboratory findings						
C3	1	–	–	1.03	0.10, 10.24	0.980
C4	1	–	–	1.003	1.001, 1.005	**0.008**
Anti-dsDNA antibody	1	–	–	1.00	0.99, 1.00	0.360
Anti-SM antibody	1	–	–	3.44	1.31, 9.51	**0.02**
IgG	1	–	–	2.06	1.30, 3.30	**0.002**
IgA	1	–	–	0.51	0.41, 0.64	**<0.0001**
IgM	1	–	–	0.71	0.36, 1.40	0.320
Albumin	1	–	–	4.51	0.64, 31.94	**0.010**

CI, Confidence Interval; FEM, Fixed-Effect Model; OR, Odds Ratio; REM, Random-Effects Model.The bold values indicate statistical significance with p < 0.05.

#### 3.3.1 Demographic characteristics

Univariate analysis for IP identified both advanced age (as continuous variable: MD = 6.14, 95%CI 4.73-7.56, P < 0.00001; as dichotomous variable: OR = 2.63, 95%CI 1.35-4.13, P = 0.003) and hospitalization duration (as continuous variable: MD = 5.56, 95%CI 4.66-6.45, P < 0.00001; as dichotomous variable: OR = 3.31, 95%CI 1.56-7.04, P = 0.002) as significant risk factors (**Figure 2**, **Table 2**). After adjustment for potential confounders, multivariate analysis confirmed age as an independent risk factor for IP (OR = 1.03, 95%CI 1.02-1.04, P < 0.00001). In contrast, hospitalization duration did not demonstrate significant independent association in the multivariate model (OR = 2.18, 95%CI 0.46-10.32, P = 0.330) (**Figure 6**, **Table 4**).

For non-infectious ILD, univariate analysis indicated that only hospitalization duration showed significant association (MD = 1.79, 95%CI 0.63-2.95, P = 0.002) (**Figure 2**, **Table 3**). However, multivariate analysis revealed more crucial information, identifying advanced age as a strong independent risk factor (OR = 1.05, 95%CI 1.04-1.07, P < 0.00001) (**Table 5**).

#### 3.3.2 Clinical features

In univariate analysis for IP, several clinical manifestations were significantly associated with increased risk, including fever (OR = 2.71, 95%CI 1.29-5.68, P = 0.008), serositis (OR = 2.79, 95%CI 2.19-3.56, P < 0.00001), multi-organ involvement (≥2 organs) (OR = 2.41, 95%CI 1.70-3.42, P < 0.00001), pulmonary involvement (OR = 2.57, 95%CI 1.57-4.21, P = 0.0002), cardiac involvement (OR = 3.97, 95%CI 2.26-6.98, P < 0.00001), and renal involvement (OR = 1.36, 95%CI 1.15-1.60, P = 0.0002) (**Figure 3**, **Table 2**). Multivariate analysis further confirmed fever (OR = 3.08, 95%CI 2.14-4.10, P < 0.00001), multi-organ involvement (≥2 organs) (OR = 4.19, 95%CI 1.29-5.76, P < 0.00001), and pulmonary involvement (OR = 3.94, 95%CI 1.42-10.89, P = 0.008) as independent risk factors for IP (**Figure 6**, **Table 4**). Conversely, photosensitivity (OR = 0.46, 95%CI 0.22-0.94, P = 0.030) and arthritis (OR = 0.69, 95%CI 0.51-0.94, P = 0.020) emerged as independent protective factors in the multivariate model (**Figure 3**, **Table 2**).

For non-infectious ILD, univariate analysis revealed a strong positive association between Raynaud’s phenomenon and ILD risk (OR = 2.62, 95%CI 1.86-3.69, P < 0.00001), whereas photosensitivity (OR = 0.46, 95%CI 0.24-0.89, P = 0.020) and renal involvement (OR = 0.62, 95%CI 0.45-0.85, P = 0.003) demonstrated protective effects (**Figure 3**, **Table 3**). Multivariate analysis confirmed Raynaud’s phenomenon as an independent risk factor (based on one study, OR = 3.62, 95%CI 1.34-9.77, P = 0.010), with its significance becoming more pronounced after multivariate adjustment (**Table 5**).

#### 3.3.3 Laboratory findings

Beyond clinical manifestations, laboratory investigations revealed distinctive risk patterns for IP and ILD. For IP, univariate analysis identified several significant associations: elevated levels of CRP (MD = 15.58, 95%CI 7.18-23.68, P = 0.0002), ESR (MD = 13.00, 95%CI 8.11-17.89, P < 0.00001), D-dimer (MD = 1.04, 95%CI 0.69-1.39, P < 0.00001), and WBC (MD = 2.40, 95%CI 0.14-4.67, P = 0.04), alongside decreased levels of IgG (MD = -0.96, 95%CI -1.76 to -0.15, P = 0.02), IgM (MD = -0.09, 95%CI -0.16 to -0.01, P = 0.02), albumin (MD = -5.57, 95%CI -7.46 to -3.68, P < 0.00001), and hemoglobin (Hb) (MD = -9.13, 95%CI -11.81 to -6.46, P < 0.00001) (**Figure 4**, **Table 2**). Multivariate analysis further identified independent predictors including CRP (OR = 1.01, 95%CI 1.00-1.01, P < 0.0001), C3 (OR = 1.39, 95%CI 1.10-2.85, P = 0.010), albumin (OR = 2.15, 95%CI 1.41-3.27, P < 0.0001), WBC (OR = 4.78, 95%CI 1.32-17.27, P = 0.020), and Hb (OR = 1.71, 95%CI 1.05-2.80, P = 0.030) (**Figure 6**, **Table 4**). Conversely, increased IgG levels exhibited an independent protective effect on IP (OR = 0.98, 95%CI 1.00-1.00, P = 0.010) and turned low IgG levels into an independent risk factor.

In contrast, ILD exhibited a different laboratory profile. Univariate analysis showed significantly higher levels of C4 (SMD = 0.24, 95%CI 0.11-0.38, P = 0.0005) and IgG (MD = 2.06, 95%CI 1.08-3.03, P < 0.0001) in ILD patients, while albumin (MD = -3.09, 95%CI -4.88 to -1.29, P = 0.0007), Hb(MD = -7.07, 95%CI -12.14 to -2.00, P = 0.006), and platelet(PLT) (MD = -31.00, 95%CI -50.87 to -11.12, P = 0.002) were significantly lower (**Figure 4**, **Table 3**). Multivariate analysis confirmed elevated C4 (OR = 1.003, 95%CI 1.001-1.005, P = 0.008) and IgG (OR = 2.06, 95%CI 1.30-3.30, P = 0.002) served as independent risk factors. In addition, anti-Sm antibody positivity became a new independent risk factor (OR = 3.44, 95%CI 1.31-9.51, P = 0.020) and increased IgA exhibited an independent protective effect (OR = 0.51, 95%CI 0.41-0.64, P < 0.00001) (**Table 5**).

#### 3.3.4 Medication use

Medication use patterns exhibited different risk patterns for IP. In univariate analysis for IP, both higher daily glucocorticoid(GC) dosage (MD = 23.77, 95%CI 5.88-41.66, P = 0.009) and antibacterial drug use (OR = 4.59, 95%CI 1.65-12.77, P = 0.004) demonstrated significant positive associations with infection risk (**Figure 5**, **Table 2**). Multivariate analysis further established antibacterial drugs (OR = 3.56, 95%CI 2.19-5.77, P < 0.00001) and immunosuppressants (OR = 5.88, 95%CI 2.74-12.62, P < 0.00001) as strong independent risk factors for IP (**Figure 6**, **Table 4**).

In contrast, for ILD, univariate analysis showed no significant associations with GC, or immunosuppressants use.

#### 3.3.5 Immunogenetic factors

Analysis of immunogenetic factors revealed a significant association with IP risk. Univariate analysis indicated that the FCGR2A HH genotype was associated with increased IP risk (OR = 2.90, 95%CI 1.09-7.68, P = 0.030) (**Table 2**). Multivariate analysis further supported this finding, confirming the FCGR2A HH genotype as an independent risk factor for IP (OR = 3.08, 95%CI 1.25-7.76, P = 0.017) (**Table 4**).

In contrast, no studies investigating immunogenetic factors in relation to non-infectious ILD risk were identified in our systematic literature search, either in univariate or multivariate analyses.

### 3.4 Subgroup analysis

**Figure 7:**
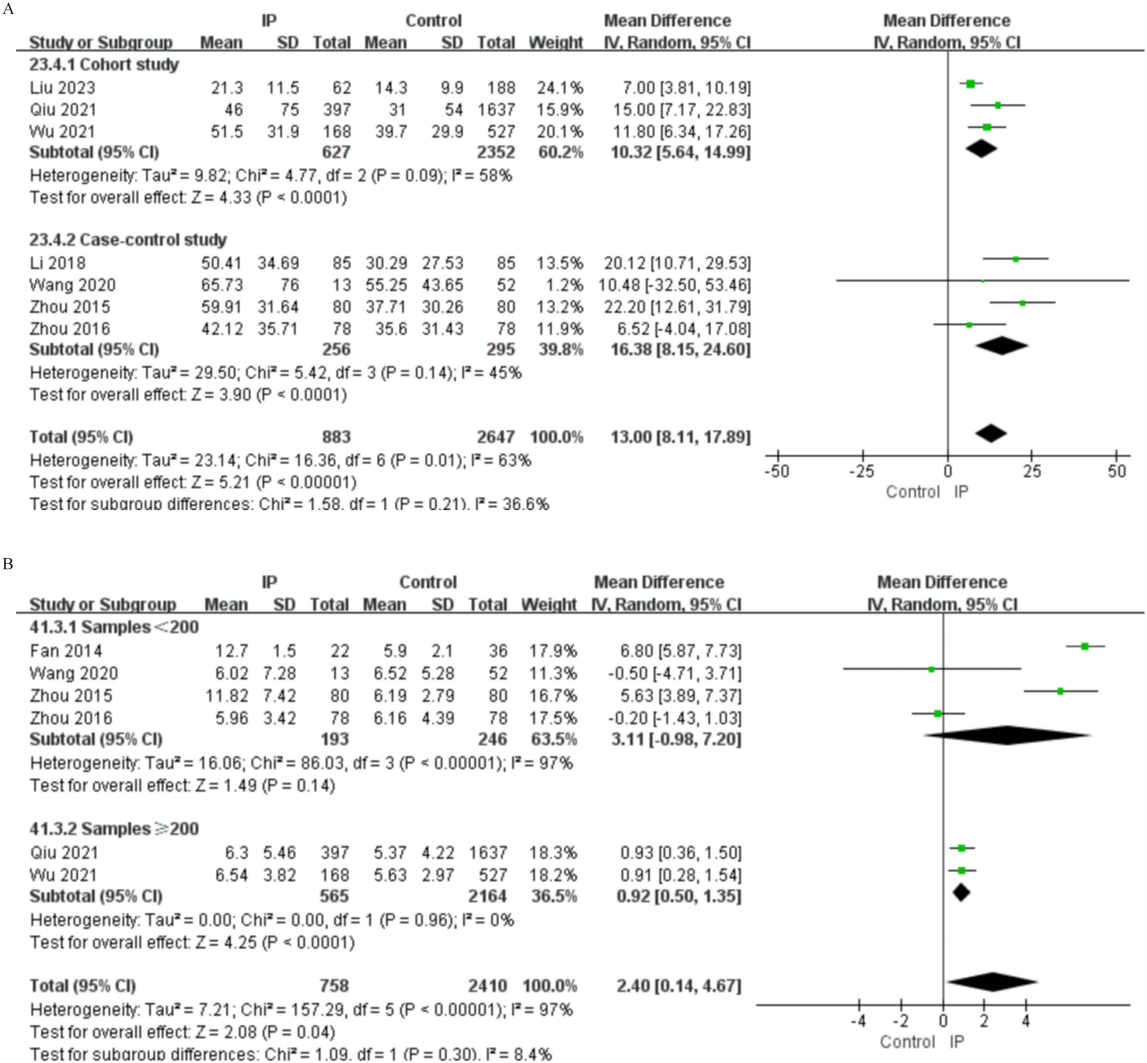
Subgroup analysis of heterogeneity sources. **(A)** ESR analysis by study design **(B)** WBC count analysis by sample size.

Subgroup analyses were also conducted according to study design for the variables with potential heterogeneous results. For ESR (7 studies in IP group), analysis by study design showed low heterogeneity among casecontrol studies (I² = 45%; MD = 16.38, P < 0.0001) and moderate heterogeneity in cohort studies (I² = 59%; MD = 10.32, P < 0.0001). The overall analysis displayed obvious heterogeneity (I² = 63%; MD = 13.00, P < 0.00001), and there was no significant difference between subgroup analyses (P = 0.210) (**Figure 7A**), which suggested that the study design was not the main cause of the between-study heterogeneity.

Furthermore, we also conducted subgroup analysis according to sample size for the 6 studies about WBC in IP group. The analysis of the studies with more than 200 participants showed no heterogeneity (I² = 0%; MD = 0.92, P < 0.0001) with statistically significant differences between groups, while the analysis of the studies with fewer than 200 participants showed considerable heterogeneity (I² = 97%; MD = 4.35, P = 0.140) with non-significant differences between groups. The overall analysis showed a mean difference of 1.90 (P = 0.040) with substantial heterogeneity (I² = 97%). The non-significant difference between the subgroups (P = 0.300) (**Figure 7B**) showed that sample size was not the main cause of between-subgroups heterogeneity.

### 3.5 Sensitivity analysis

In addition, we compared the pooled OR values obtained from both FEM and REM to assess the robustness of the identified independent risk factors for IP in SLE patients. As shown in **Table 6**, the results showed that the two analytical methods yielded remarkably similar results for all evaluated factors, and no reversal of statistical significance or effect direction was observed for any risk factor. The similarity of the results from the two statistical models further showed that the analytical results were low sensitive.

**Table 6:** Comparison of calculation results of FEM and REM to independent risk factors for IP in SLE.

Risk factors	FEM combined OR (95%CI)	REM combined OR (95%CI)
Age	1.03 (1.02, 1.04)	1.03 (1.02, 1.04)
Pulmonary involvement	3.24 (1.88, 5.59)	3.94 (1.42, 10.89)
CRP	1.01 (1.00, 1.01)	1.01 (1.00, 1.02)
WBC	4.78 (1.32, 17.27)	5.27 (1.12, 24.80)
Antibacterial drugs	3.56 (2.19, 5.77)	3.56 (2.19, 5.77)
Immunosuppressants	5.88 (2.74, 12.62)	5.88 (2.74, 12.62)

CI, Confidence Interval; FEM, Fixed-Effect Model; OR, Odds Ratio; REM, Random-Effects Model.

### 3.6 Publication bias

**Figure 8:**
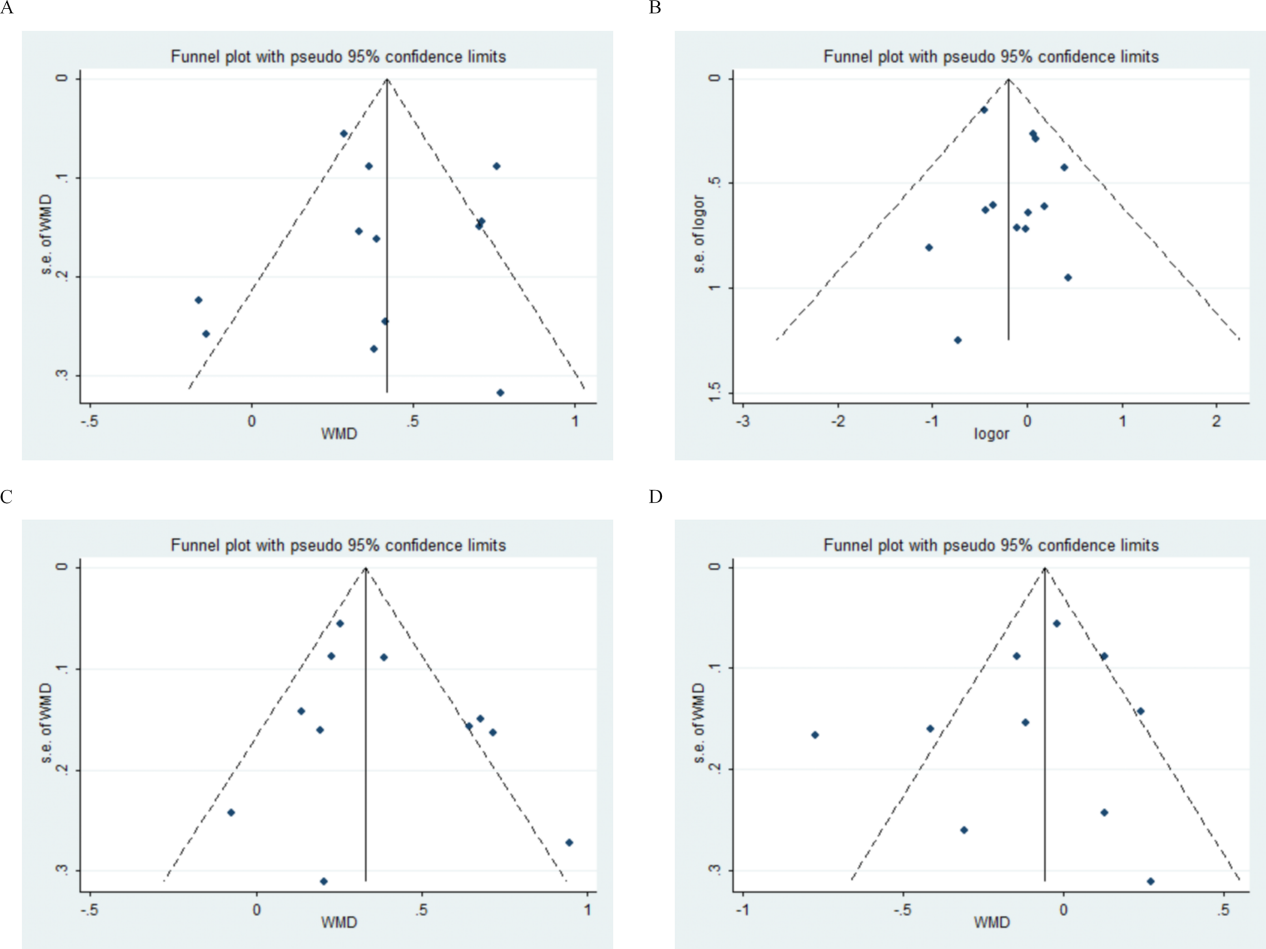
Funnel plots for assessment of publication bias. **(A)** Age **(B)** Gender **(C)** ESR **(D)** C3.

For the assessment of publication bias, we chose four representative factors for bias inspection, age and gender for demographic factors and ESR and C3 for laboratory factors.The funnel plots for the bias inspection of the above four factors are shown in **Figure 8**, and no significant skewness was found. The result of inspection was further supported by Egger’s regression test, and the obtained p-value was not significant (age: P = 0.932; gender: P = 0.372; ESR: P = 0.287; C3: P = 0.525), which indicated that there was no significant publication bias for all of the selected factors.

## 4 Discussion

This meta-analysis aims to address the first comparison of risk factors for IP and non-infectious ILD in patients with SLE. To support the interpretation of our results, we build an evidence stratification framework where we prioritize multivariate-adjusted associations (**Table 7**). By integrating evidence under this stratification framework, we show that IP and ILD display profoundly different risk profiles. This stratification of evidence not only enhances our understanding of the distinct pathogenic mechanisms of these two conditions but also offers an essential evidence basis for the development of targeted clinical strategies, allowing for a critical appraisal of which factors are most likely to be causally relevant and immediately actionable.

**Table 7:** Summary of evidence stratification framework for risk factors of pulmonary complications in SLE.

Evidence stratification	IP	Non-infectious ILD
Robust independent risk factors	age, pulmonary involvement, CRP, WBC, immunosuppressants antibacterial drugs	None identified yet
Preliminary independent risk factors	fever, multi-organ involvement, C3, Low IgG, hypoalbuminemia, low Hb, FCGR2A HH genotype	age, Raynaud's phenomenon, C4, anti-Sm antibody, high IgG, hypoalbuminemia
Potential risk factors	hospitalization duration, serositis, cardiac involvement, renal involvement, elevated SLEDAI, GC	hospitalization duration, hypoalbuminemia, decreased Hb
Protective factors	photosensitivity, arthritis	High IgA

ILD, Interstitial lung disease; IP, Infectious pneumonia.

### 4.1 IP: The dual drivers of immunosuppression and systemic inflammatory burden

Risk factors for IP can be summarized in two underlying drivers: acquisitive immunodeficiency and systemic inflammation/disease burden. One of the most important findings of this study is the identification of iatrogenic immunosuppression as a key driver of IP. The class of immunosuppressants and antibacterial agents (two robust independent predictors), identified as the strongest and most consistent predictors of IP risk highlight a fundamental clinical conundrum in SLE management.

Immunosuppressants regulate autoimmune activity by inhibiting the proliferation and function of T and B lymphocytes, thereby reducing autoimmunemediated damage; however, this immunomodulatory environment also predisposes patients to opportunistic infections (31, 32). However, not all immunosuppressants have the same potential to induce infection. As an alkylating agent, cyclophosphamide non-selectively and significantly impairs cellular and humoral immune responses to bacteria, viruses and fungi (33, 34). In contrast, mycophenolate mofetil has a more selective inhibitory effect on lymphocytes, leading to an infection risk profile that is distinct from cyclophosphamide (35, 36); Indeed, several studies have reported that patients treated with mycophenolate mofetil are associated with a lower risk of infections when compared with cyclophosphamide (37). In addition, antimalarial agents such as hydroxychloroquine may protect against infections through pH-dependent iron deprivation and increased lysosomal pH. Interestingly, hydroxychloroquine’s antifungal properties may provide protection against fungal infections (38). When used in SLE management, therefore, immunosuppressants should be considered not only for their immunosuppressive effectiveness, but also for their respective infection risk profiles, which will be important in individualized treatment.

More importantly, the usage of antibacterial drugs was found to be a strong independent risk for IP. This finding may be due to their potential to disrupt the normal microbiota, which may increase colonization of resistant pathogens and indirectly increase IP risk in SLE patients (39). In addition, antibacterial drugs can serve as an important indicator reflecting the underlying high-risk status. Specifically, antibacterial prescriptions reflect two important clinical scenarios (1): The treatment of infection, which demonstrates that the patient’s immune defenses have already been impaired (40); and (2) prophylactic administration in immunosuppressed patients, which means that clinicians have recognized that the patient is at extremely high risk for infection due to either highly active underlying disease or intensive immunosuppressive therapy (41). Therefore, the usage of antibacterial drugs can act as a composite measure reflecting both severe immunosuppressive status and preexisting infection burden. The strong significance of this association remained after adjusting for other factors in our multivariate analysis, further validating the value of this important variable in predicting IP risk in SLE patients.

Beyond immunosuppression, systemic inflammation and significant underlying disease burden provide grounds for IP. Advanced age emerged as another robust predictor, as older patients with SLE will present with immunosenescence, including thymic involution and poor T-cell function. This immunosenescence associated with advanced age, together with a higher prevalence of comorbidities (e.g., diabetes mellitus and chronic kidney disease), will lead to an increased burden of infection (42–44). Meanwhile, the levels of CRP and WBC maintained independent predictive value in multivariate analyses, indicating that an active state of systemic inflammation may also contribute to IP risk (45, 46). It is worth noting that our meta-analysis measured the increased CRP level of IP patients, which was found to be 15.58 higher than in SLE controls. Although there is clearly a large degree of heterogeneity which is probably due to variability in the extent of infection and its timing of observation, the CRP values of IP groups often surpassed 10 mg/L. Hence, a CRP level over 10mg/L in an SLE patient should effectively raise a red flag against the possibilities of a superimposed infectious pneumonia, and it should be actively investigated in clinical practice. This is data-driven threshold that is consistent with clinical general practices of identifying major inflammation. On the same note, an increase in the WBC level above the normal levels is a supportive evidence. It is very important to interpret these laboratory findings to the entire clinical context with neither being pathognomonic. Notably, we confirmed that pre-existing pulmonary involvement maintained an important strong independent risk factor for IP despite moderate heterogeneity caused by differences in definitions of pulmonary pathology among studies. This strong association has clear clinical rationale. Pre-existing pulmonary involvement (e.g., pneumonia, pleural effusion, or alveolar hemorrhage) can directly impair the structural defenses of the lungs and provide favorable conditions for the colonization and multiplication of pathogens (47).

In addition, several preliminary independent predictors delineate the clinical profile of SLE patients at high risk for IP. Fever represents a frequent manifestation of infection; however, it is also an important marker of high disease activity in SLE. It reflects a highly inflammatory state and cytokine storm that can interfere with normal functions of effector immune cells and thus facilitate pathogen invasion (48). Multi-organ involvement (≥2 organs) is a strong predictor of global disease severity and activity. In animal studies, higher SDI scores were associated with infections in SLE (49). This extensive multi-organ involvement generally requires more potent or prolonged immunosuppressive therapy, while also reflecting reduced physiological reserve and organ function, which sum total the increased risk for infection (17, 50).

Concurrently, hypoalbuminemia reflects poor nutritional state and continued inflammatory consumption, which affects both tissue repair and synthesis of immune proteins. Anemia in SLE, frequently due to immune-mediated destruction, may induce hypoxia states at potential foci of infection locally through immune-mediated destruction, thus increasing susceptibility to acute infections (51). In aggregate, these factors describe a patient with active disease and reduced physiological reserve, who presents increased vulnerability to infectious attack.

Genetic predisposition represents an important modulator of infection risk in SLE. The FCGR2A HH genotype, with its reduced FcγRIIa receptor affinity for CRP, impairs phagocytic clearance of Streptococcus pneumoniae and thereby increases susceptibility to IP in this patient population (17). This molecular phenotype provides a model for understanding the heightened risk for infection in SLE patients with FCGR2A HH genotypes and thus a rationale for closer attention to infection surveillance in these high-risk patients. In sum, the development of IP in SLE patients represents a synergistic combination of immunosuppression, barrier compromise and inflammation throughout the body.

It is interesting that certain factors, such as renal involvement, although significantly associated with IP risk in univariate analysis, lost significance after adjustment for other variables in multivariate models. Thus, renal involvement in SLE patients likely represents a surrogate marker for global disease severity and immunotherapy in SLE patients and not a direct independent cause of infection. In clinical practice, although the presence of renal involvement should raise the index of suspicion for increased IP risk in a given SLE patient, clinicians should appreciate that this risk is mediated largely through the associated immunotherapy and global immune state rather than attributing this risk directly to renal involvement per se.

### 4.2 Non-infectious ILD: The central role of specific autoimmunity and vascular pathology

Raynaud’s phenomenon emerged as the most distinctive and significant clinical predictor for ILD. As shown in Liu et al. reported that the rates of serositis, positivity of anti-Sm antibody and anti-U1-nRNP antibody in SLE patients with Raynaud’s phenomenon as initial manifestation were significantly higher than that in SLE patients with Raynaud’s phenomenon not as initial manifestation (52). It is well established that vasospasm, endothelial injury and sustained microvascular dysfunction may play a key role in the initial event and further development of pulmonary fibrosis (53), which connects systemic vascular pathology and specific lung injury.

At the serological level, the co-existence of anti-Sm antibody positivity and elevated IgG level also demonstrate the central role of B-cell hyperactivation and specific autoimmunity in the process of ILD development. In line with this concept, Gong et al. found that reduced levels of IgG and switched memory B-cells (OR = 1.27, P = 0.0029) as well as IgD- CD38dim B-cells (OR = 1.08, P = 0.0449) were significantly increased in IPF patients (54).

In this study, we observed that pathophysiologically, serum IgG levels have fundamentally different association patterns between SLE-IP and SLE-ILD. That is, low IgG levels is an independent risk factor for IP; while elevated IgG levels show significantly associated with the development of non-infectious ILD. This paradoxical relationship provides us with important immunological information for understanding the pathogenesis of these two PC. From an immunological defense standpoint, IgG is the main component of humoral defense and plays an indispensible role in eliminating extracellular pathogens. Reduced serum IgG level is directly correlated with the state of humoral immunodeficiency (55). This immunodeficiency may be caused by primary B-cell dysfunction induced by underlying diseases or by immunosuppressive therapies targeting B cells (56). Clinically, low IgG status is associated with insufficient production of neutralizing antibodies and opsonins, greatly impairing the clearance of respiratory pathogens and increasing susceptibility to IP (57). In contrast, in the pathogenesis of ILD, high IgG level is not associated with effective immune defense but with serological effluvia of hyperactive autoimmunity. The increased fraction of IgG may contain autoantibodies targeting lung tissue antigens and induce chronic tissue inflammation and fibrotic progression through the following mechanisms: formation of immune complexes, activation of complement system, and recruitment of inflammatory cells (58). This discovery is clearly elucidative of the distinct immunopathogenesis of SLE-IP versus SLE-ILD: the former mainly results from suboptimal immune defense, namely, decreased ability to eliminate exogenous pathogens; the latter mainly results from suboptimal immune tolerance, namely, excessive attack on self-tissues. This distinction holds important implications for clinical practice: the serum IgG level of SLE patients should be assessed in specific clinical scenarios - maintaining an adequate level of serum IgG is indispensible to prevent and control IP; suppressing the excess B-cell activation and autoantibody production becomes the main therapeutic goal in ILD.

Interestingly, we found that elevated C4 levels was an independent risk factor for SLE-ILD, which provides a striking counterexample to the expected pattern of systemic complement activation and consumption commonly seen in active SLE. This paradoxical finding suggests the existence of atypical complement dynamics in SLE-ILD. As demonstrated in Liu et al.’s study, in idiopathic pulmonary fibrosis, the complement system does not present the consumption-induced hypofunction commonly seen in some classic autoimmune diseases but exists in a state of hyperactive activation (59). This activation is manifested not only by the upregulation of several complement activation markers in the system circulation but, more importantly, by the local complement system activation in lung tissue that drives inflammatory and fibrotic processes (although the specific mechanisms remain incompletely elucidated). Consistent with this concept, Kulkarni et al. have demonstrated the deposition of complement activation products in lung tissue (60).

Advanced age and hypoalbuminemia were identified as independent risk factors for ILD, consistent with their roles in IP. These two factors may serve as markers of systemic inflammation and overall disease severity but are unlikely to be drivers of the ILD pathogenesis. This observation further highlights the complexity of the relationships among systemic factors, organ-specific risk factors, and ILD risk.

### 4.3 Protective factors and explanations for disease heterogeneity

The identification of protective factors provides important viewpoints to explain disease heterogeneity in SLE. Interestingly, our study demonstrated that arthritis and photosensitivity, two typical clinical manifestations, were independently associated with decreased risk of IP. This unexpectedly paradoxical result may indicate the existence of different SLE endotypes: an “external phenotype” focusing on joints and skin with tissue localized inflammation may be totally different from immune phenotypes in developing severe visceral infections such as IP which are associated with extreme B-cell depletion and/or neutrophil dysfunction (61).

The protective effect of increased IgA level on IP may imply that mucosal immunity may have a special protective effect against pulmonary fibrosis although the underlying mechanisms are still unclear. One possible explanation may lie in the fact that IgA, with its anti-inflammatory properties and ability to modulate neutrophil function (62), may play a protective role by preventing the chronic inflammatory environment from inducing fibrosis. This hypothesis deserves further exploration in future studies.

### 4.4 Clinical implications and limitations

Stratified evidence profile exhibited by this study has important immediate clinical relevance. Based on our findings, clinical proactive stratified risk stratification and corresponding management for PC in SLE are now possible. Distinct risk factor profiles for IP and ILD support the idea of precision medicine: For the prevention of IP, special caution should be paid to patients with advanced age, ongoing immunosuppressive therapy, high inflammatory markers (CRP, WBC), low IgG level, or even previous pulmonary involvement. These patients may require intensified infection surveillance and prophylactic measures as well as updated immunization. For the early detection of ILD, patients with advanced age, Raynaud’s phenomenon, positive anti-Sm antibody, or high IgG level should undergo pulmonary function tests and high resolution CT screening even if they have no respiratory symptoms.

Despite these clinical implications, several important limitations should be noted when interpreting our findings. First, the number of studies available for multivariate meta-analysis for ILD was severely limited. Many preliminary independent associations are based on a single study and cannot be evaluated for consistency and precision using traditional meta-analytic pooling. We instead used a structured narrative summary to present existing evidence in a transparent manner while acknowledging that future validation is needed. Second, there was considerable heterogeneity for some factors (eg, pulmonary involvement in IP), which may reflect differences in study populations or variable definitions of risk factors or clinical settings. We used random-effects models and subgroup analyses when possible, but residual heterogeneity precludes confident interpretation of pooled results. Third, uncontrolled confounding in the original observational studies cannot be excluded. Despite focusing on multivariate-adjusted estimates, unmeasured confounding factors might still influence the observed associations.

## 5 Conclusion

In conclusion, by employing a first direct comparative design and a novel evidence stratification framework, this study elucidates fundamentally distinct risk architectures for infectious pneumonia and non-infectious interstitial lung disease in SLE. IP is predominantly immunosuppression and global inflammatory effects of the disease, whereas ILD is comprised of unique features of active autoimmunity and vascular pathology. The stratified evidence synthesized in this study offers the basis for a framework to reconsider how we view pulmonary complication risk in SLE. Prevention and diagnostic strategies can be tailored to each patient`s disease profile. Future studies should focus on validation of these multivariate relationships using larger prospective studies to confirm these relationships and explore biological mechanisms.

## Data Availability

All data analyzed in this meta-analysis are available from published studies cited in the references, with **Supplementary Material** and protocols accessible via PROSPERO (CRD420251020965).
