# Supplementary material for: Risk factors for pulmonary complications in systemic lupus erythematosus: a meta-analysis of infectious pneumonia and interstitial lung disease
Source: Front Immunol. 2026 Mar 18;17:1758316. doi: 10.3389/fimmu.2026.1758316 (PMC13038906; doi:10.3389/fimmu.2026.1758316)
Supplement: Supplementary file 1 [file Table1.docx]

**Supplementary Table 1**. Search strategy in PubMed Database

| Number | Search terms |
| --- | --- |
| #1 | Lupus erythematosus, systemic[Mesh Terms] |
| #2 | Lupus erythematosus disseminatus[Title/Abstract] |
| #3 | SLE[Title/Abstract] |
| #4 | #1 OR #2 OR #3 |
| #5 | Pneumonia[Mesh Terms] |
| #6 | Infectious Pneumonia[Title/Abstract] |
| #7 | Lung infection[Title/Abstract] |
| #8 | Interstitial lung disease[Title/Abstract] |
| #9 | #5 OR #6 OR #7 OR #8 |
| #10 | Risk Factors[Mesh Terms] |
| #11 | Population at Risk[Title/Abstract] |
| #12 | Health Correlates[Title/Abstract] |
| #13 | Risk Score[Title/Abstract] |
| #14 | Etiology[Title/Abstract] |
| #15 | Associated Factor[Title/Abstract] |
| #16 | #10 OR #11 OR #12 OR #13 OR #14 OR #15 |
| #17 | #4 AND #9 AND #16 |
